# Internal Auditory Canal Glioneural Hamartoma: A Rare Mass Masquerading as a Vestibular Schwannoma

**DOI:** 10.7759/cureus.37361

**Published:** 2023-04-10

**Authors:** Daphne Li, Rachyl Shanker, Ewa Borys, John P. Leonetti, Douglas E Anderson

**Affiliations:** 1 Neurosurgery, Loyola University Medical Center, Maywood, USA; 2 Pediatric Neurosurgery, Advocate Lutheran General Hospital, Park Ridge, USA; 3 General Surgery, University of Illinois Chicago, Chicago, USA; 4 Neuropathology and Renal Pathology, Loyola University Medical Center, Maywood, USA; 5 Neuropathology and Renal Pathology, Edward Hines Jr. Veterans Administration Hospital, Hines, USA; 6 Otolaryngology, Loyola University Medical Center, Maywood, USA; 7 Neurological Surgery, Loyola University Medical Center, Maywood, USA; 8 Neurological Surgery, Edward Hines Jr. Veterans Administration Hospital, Hines, USA

**Keywords:** internal auditory canal, cerebellopontine angle, intracanalicular mass, glioneural heterotopia, glioneural hamartoma

## Abstract

Glioneural hamartomas are exceedingly rare lesions. When localized to the internal auditory canal (IAC), they can cause symptoms referrable to seventh and eighth cranial nerve compression. Here, the authors present a rare case of an IAC glioneural hamartoma. A 57-year-old male presented for evaluation of presumed *intracanalicular vestibular* schwannomas found on work-up of dizziness and progressive right-sided hearing loss. Surgical intervention pursued progressive symptoms and new onset headaches. The patient underwent uncomplicated retrosigmoid craniectomy for gross total resection. Histopathological evaluation revealed a glioneural hamartoma. A MEDLINE database search used the terms' cerebellopontine angle' OR 'internal auditory canal' AND 'hamartoma' OR 'heterotopia'. Clinicopathological characteristics and outcomes of the present case were compared to those in the literature. The literature review yielded nine articles describing 11 cases (eight females, three males; median age 40 years, range 11-71) of intracanalicular glioneural hamartomas. Patients most commonly presented with hearing loss and were presumed to have a diagnosis of vestibular schwannoma before histologic diagnosis. Glioneural hamartomas are rare lesions that may be found in the IAC. Although benign, they may be safely resected for cranial nerve function preservation goals with a low risk of recurrence.

## Introduction

Glioneural hamartomas or heterotopia are exceedingly rare lesions that may arise in the internal auditory canal (IAC) and masquerade as a more common pathology [1]. Most masses in the IAC constitute vestibular schwannomas (VS), followed by meningiomas [2]. Similar to these more common lesions, glioneural hamartomas present commonly with symptoms of mass effect on the seventh and eighth cranial nerve (CN) complex. Preoperative imaging modalities, such as computed tomography (CT) or magnetic resonance imaging (MRI), may not help differentiate these masses from their more common counterparts. However, glioneural hamartomas are less likely to demonstrate post-contrast enhancement. Although abnormal, these are generally benign lesions that are slow growing and rarely recur after resection. Surgical intervention is often pursued goals of CN function preservation. Here, the authors present a rare case of glioneural hamartoma in a middle-aged man who was presumed to have a VS managed with surgical intervention. The clinical and pathological case characteristics are compared to those currently available in the literature to consolidate the current fund of knowledge on this rare clinical entity.

## Case presentation

Clinical presentation

**Figure 1:**
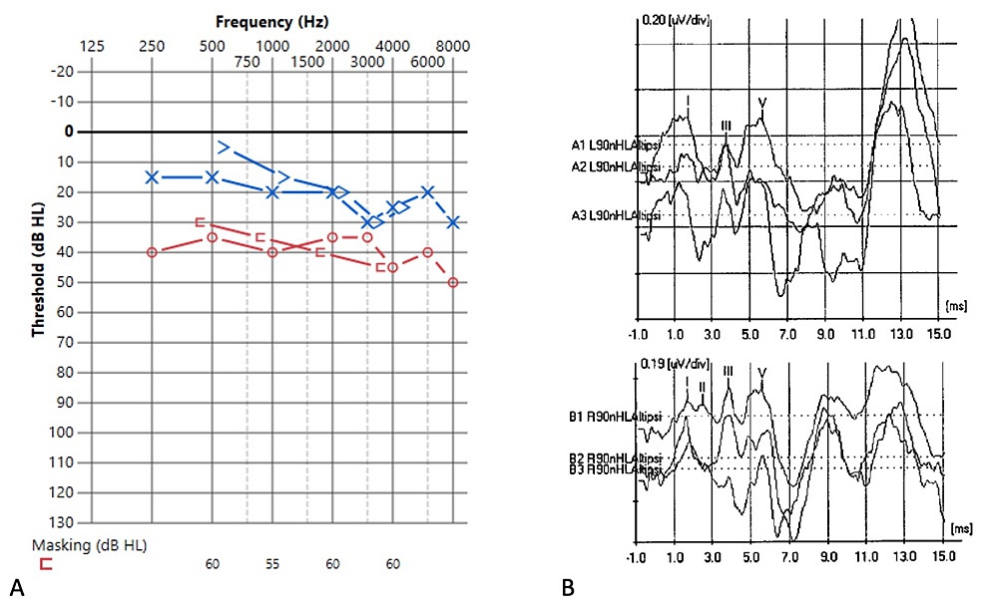
Preoperative audiogram (A) and auditory brainstem responses (B) Audiogram recordings demonstrating moderate sensorineural hearing loss 250-8000 Hz on the right ear. The patient had normal hearing in the left ear across tested frequencies 250-800 Hz, with a mild sensorineural notch at 3000-4000 Hz. Word recognition scores were excellent when tested at a much higher than the normal conversational level in the right (96%) and normal conversational level in the left ear (96%).

**Figure 2:**
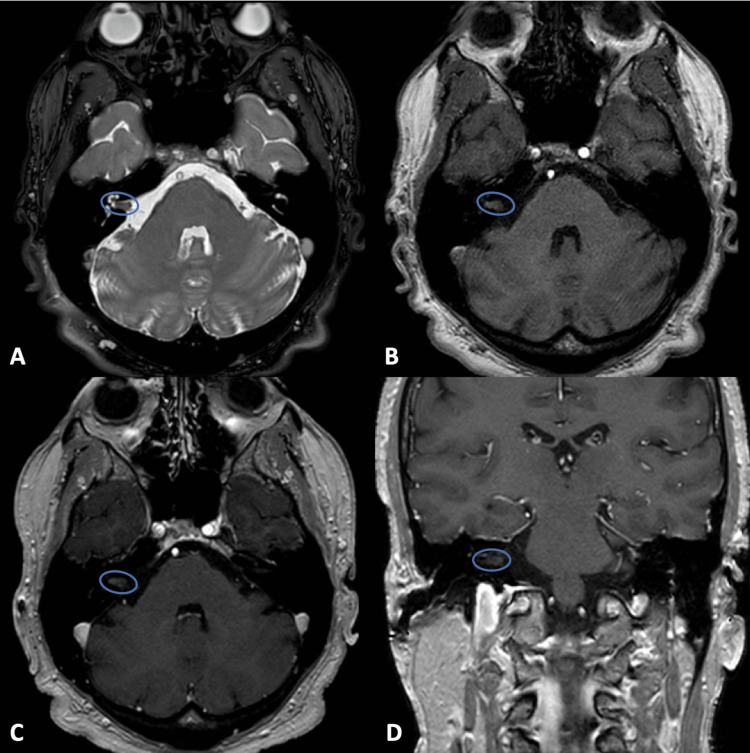
Preoperative magnetic resonance imaging (MRI) A-C: Axial T2-weighted (A) and T1-weighted fast field echo pre-contrast (B) imaging, and axial (C) and coronal (D) T1 post-contrast imaging demonstrating a T2 and T1 isointense lesion (blue circle) without contrast enhancement located anteriorly in the internal auditory canal. No evidence of significant bony remodeling or expansion of the IAC.

A 57-year-old male presented to the otolaryngology and neurosurgery clinic after evaluation of dizziness and progressive right-sided hearing loss revealed an intracanalicular mass. The patient was found to have slight asymmetric sensorineural hearing loss on the audiogram (Figure 1). His neurologic exam was otherwise unremarkable, and the MRI demonstrated a small, 0.75x0.32cm, non-enhancing mass in the IAC (Figure 2). The mass was presumed to be a VS during preoperative work-up, and recommendations were made for radiographic surveillance versus radiation or surgery. After four months of observation, the patient returned to the clinic and elected for resection of his tumor, given his progressive symptoms and new onset headaches. The risks and benefits of all management options were discussed with the patient before pursuing a surgical intervention.

Surgical intervention

**Figure 3:**
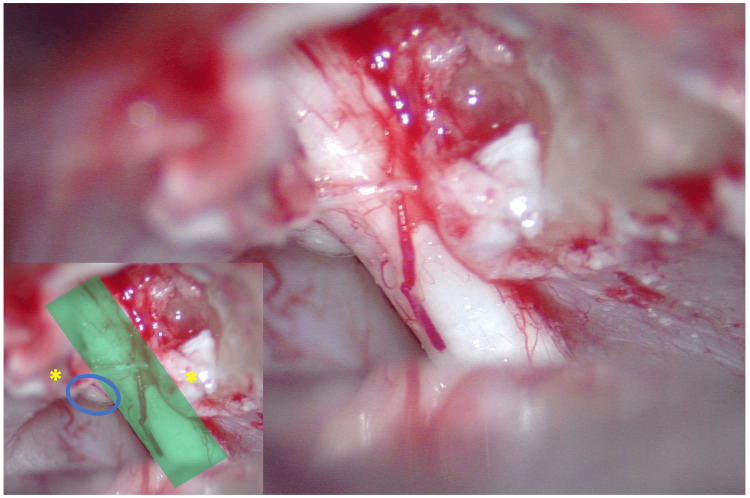
Intraoperative microscope photo (annotated inset) A metal retractor lies medially over the cerebellum at the image's inferior aspect. At the same time, the porus acusticus has been drilled to expose the contents of the internal auditory canal (IAC, seen at the superior aspect of the image). Viewed posteriorly through a retrosigmoid approach, the dura lining the IAC (yellow asterisk) has been sectioned and reflected superiorly and inferiorly to expose the seventh and eighth nerve complex (green shading). A portion of the tumor (blue oval) is located ventrally and superiorly in the IAC. IAC: internal auditory canal

The patient underwent an elective right retrosigmoid craniectomy to resect his IAC mass. Intraoperative neuromonitoring with somatosensory evoked potentials was employed along with electromyography of CN VII. Auditory brainstem responses were monitored throughout the procedure. The CN VII/VIII complex was isolated before drilling the porus acusticus. Direct nerve stimulation demonstrated intact CN VII/VIII with evidence of the tumor located anterosuperiorly to the facial and cochlear nerve (Figure 3). The tumor was firm and appeared grossly similar to its surrounding tissues. Using careful microdissection, the tumor was able to be removed en bloc. Due to anatomic constraints, the lateral aspect of the IAC was not able to be thoroughly examined. Still, the size and appearance of the specimen were consistent with a gross total resection. Dura and superficial tissues were closed in standard fashion. There were no intraoperative complications, and the patient was extubated without issue.

Histology

**Figure 4:**
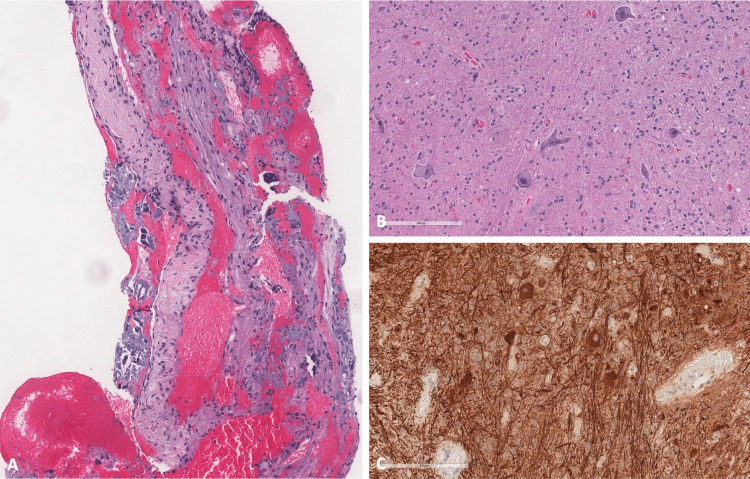
Histopathology A: Low power H&E section. B: High power H&E section. C: Neurofilament stain. Sections demonstrating fragments of nerve, large neuronal cell bodies, some in clusters and intervening axons. Findings are consistent with glioneural hamartoma.

Histopathological evaluation revealed a glioneural hamartoma (or heterotopia) (Figure 4). Histologic sections demonstrated a portion of glioneuronal tissue consistent with hamartomatous brain tissue. Small fragments of nerve were included. Neurofilament stain highlighted large neuronal cell bodies, some in clusters and intervening axons.

Postoperative course

The patient tolerated the procedure well despite mild postoperative nausea and persistent dizziness. He had normal facial function (House-Brackmann 1). He was discharged home on postoperative day four with recommendations for home therapy services. Postoperative imaging was consistent with gross total resection of mass without recurrence. Audiology evaluation demonstrated slight worsening in hearing loss on the right side, amenable to hearing aid placement. He was last seen in the clinic 2 years after surgery with normal facial nerve function and no evidence of tumor recurrence.

## Discussion

**Figure 5:**
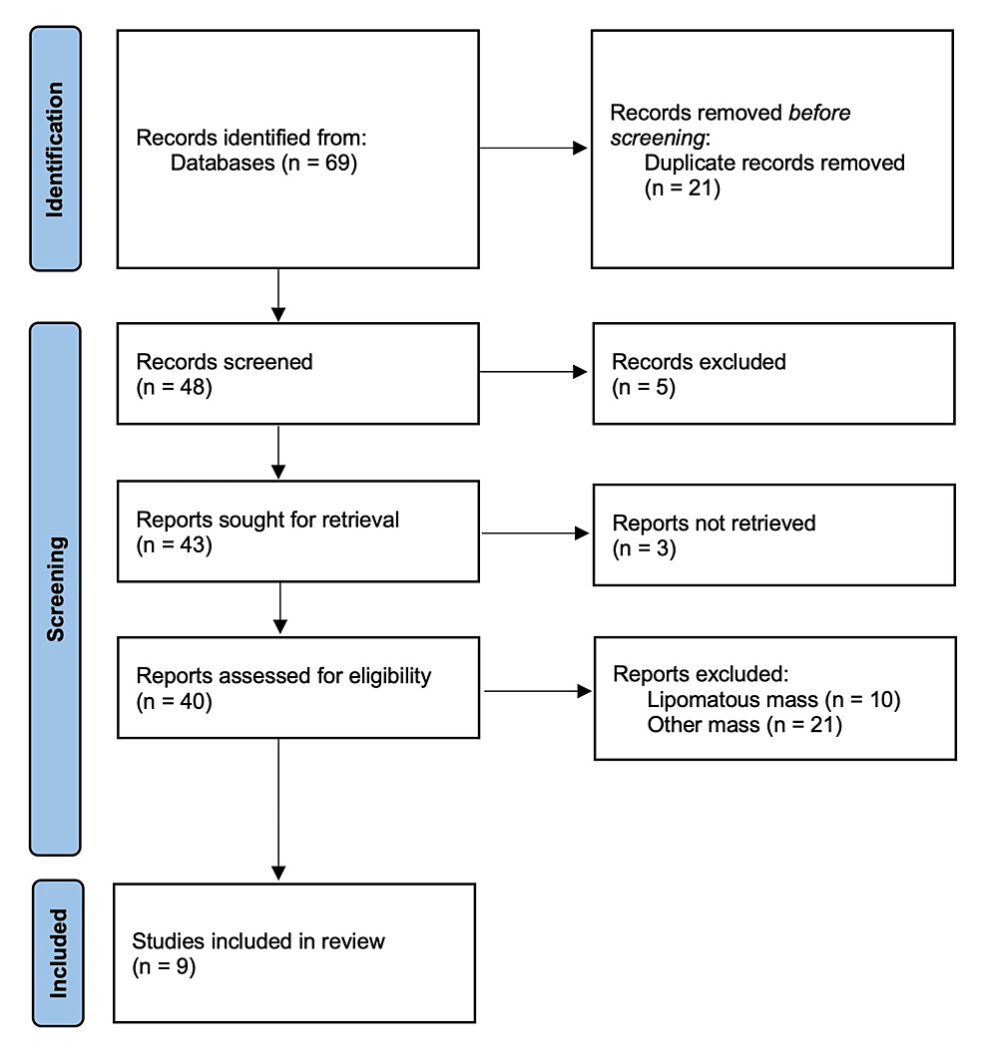
PRISMA flow diagram of citations identified and evaluated on a literature review of glioneural hamartomas of the internal auditory canal. Page MJ, McKenzie JE, Bossuyt PM, Boutron I, Hoffmann TC, Mulrow CD, et al. The PRISMA 2020 statement: an updated guideline for reporting systematic reviews. BMJ 2021;372:n71. doi: 10.1136/bmj.n71

A computerized search of the MEDLINE database (Pubmed) through January 2023 was conducted using the search terms' cerebellopontine angle' OR 'internal auditory canal' AND 'hamartoma OR 'heterotopia' (Figure 5). Articles were included if reports described cases of glioneural hamartomas or heterotopia that are located primarily in the IAC. Articles were excluded if they were not published in English literature, full-text articles could not be reviewed, or case details were not provided.

Our literature review yielded nine articles from 1980 to 2018 describing 11 patients with glioneural hamartomas arising in the IAC. A summary of patient demographics and case characteristics is found in Table 1. There were three males (27%) and eight females (73%), with a mean age of 41.4 years old (median: 40, range: 11-71). Patients commonly presented with hearing loss (n = 7/8) [3-8], hyperacusis or tinnitus (n = 3/8) [6,7], vertigo (n = 2/8) [3,6], CN VII palsy (n = 2/8) [4,6], CN V paresthesias (n = 2/8) [4,9], or headaches (n= 2/8) [4,7]. Three cases described patients with incidentally discovered lesions on autopsy [10,11].

**Table 1:** Systematic review of internal auditory canal glioneural hamartomas reported in the literature Abbreviations: ABRs = auditory brainstem response, BAERs = brainstem auditory evoked repsonses, CN = cranial nerve, CPA = cerebellopontine angle, CT = computed tomography, dB = decibels, IAC = internal auditory canal, F = female, GTR = gross total resection, HB = House-Brackmann, HL = hearing loss, L = left, M = male, m = month, MRI = magnetic resonance imaging, N/A = not applicable, NS = not specified, R = right, SNHL = sensorineural hearing loss, y = year

Author & Year	Age (y) / Gender	Presentation	CT/MRI Appearance	Audiogram Findings	Preliminary Diagnosis	Management	Intraoperative Findings	Outcome
Babin et al., 1980 [10]	43 / M	No history of vertigo	N/A	N/A	N/A	N/A	Autopsy - R IAC compact fusiform mass, closely applied to glial sheaths of the cochlear nerve.	N/A
	36 / F	NS	N/A	N/A	N/A	N/A	Autopsy - 10mm nodule intimately associated with CN VIII at R CPA	N/A
Ho et al., 1981 [11]	67 / M	NS	N/A	N/A	N/A	N/A	Autopsy - 2x3mm leptomeningeal nodule at CPA near the exit of CN VIII. No association with the medulla or CN.	N/A
Palmer et al., 1996 [7]	71 / F	6y history of intermittent vertigo, subjective R HL mild	MRI: Small enhancing R IAC mass extending into CPA	Normal	NS	Observation for 4m followed by R posterior fossa craniectomy for interval increase in size	A tumor arose from the cochlear nerve, GTR.	R CN VII palsy that improved over 1y, Postoperative audiogram near normal, no recurrence after 20m
Hung et al., 1997 [6]	40 / F	5y of progressive R HL	MRI: 0.5cm enhancing mass in IAC	Moderate-severe SNHL	VS	Suboccipital approach for hearing preservation	Dilated IAC rostrocaudally by laterally located mass from an acoustic portion of CN VIII complex, displaced and attenuated vestibular nerve; GTR with the sacrifice of acoustic nerve, but the preservation of CN VII	complete preservation of CN VII function
Carlvaho et al., 2003 [4]	40 / M	1.5y of progressive R HL and tinnitus, hyperacusis on R	MRI/CT: enhancing lesion inside IAC	30dB loss of speech discrimination	VS	Lateral suboccipital retrosigmoid craniectomy	Hard tumor and infiltrated both facial and cochlear nerves, no plane between tumor and nerves, marked sensitivity to manipulation with ABRs; partial tumor resection to preserve nerve function	Postop CN VII preservation, audiogram displayed additional HL 10dB between 1-3kHz; 1.5y without tumor regrowth
	39 / F	3y HA and progressive L HL, light L hyperacusis	MRI: enhancing IAC mass	HL 30dB at 1-3kHz	VS	Suboccipital retrosigmoid approach	Hard tumor with severe flattening of cochlear nerve with infiltration of vestibulocochlear nerves; early loss of BAERs; GTR with a sacrifice of vestibulocochlear nerves; facial nerve preserved	Normal facial function and L HL, 3y follow-up without recurrence
Goda et al., 2003 [5]	11 / F	3y L HL and L facial palsy, followed by sudden vomiting, HA, and hypesthesia V1-3	CT: dilated IAC with non-enhancing mass protruding into CPA	flat SNHL and neg ABR	VS	L suboccipital craniectomy	Partial resection of mass (dark red) filled canal involved nerves	No improvement of preoperative symptoms with stable small residual mass in IAC at 10y follow-up MRI without recurrence
Ahmad et al., 2010 [3]	49 / F	>4y L HL and vertigo since 12 years old, occasional tinnitus followed by eventual L facial weakness	MRI: 5x3mm enhancing mass on IAC segment of CN VIII	normal R; mild to moderate SNHL (72% speech discrimination) on L	VS or CN VII neuroma	Observation for 21 months followed by L trans labyrinthine approach for resection due to new onset L facial weakness	Lesion located on the superior vestibular nerve, extending inferiorly to the area of cochlear modiolus	L HB 1-2 facial nerve palsy at 5m follow up
Rizk et al., 2013 [8]	31 / F	Sudden L HL 6m	MRI: non-enhancing 5x9mm IAC mass displacing facial and cochlear nerves	High-frequency, moderate to severe SNHL with normal discrimination, slightly prolonged wave 1-5 and 3-5 latency	NS	Middle fossa approach craniotomy for hearing preservation	Firm lesion difficult to dissect from surrounding nerves, resection with preservation of nerves	NS
Peris-Celda et al., 2018 [9]	29 / F	Intermittent facial numbness	MRI: 4x6mm oval minimally enhancing mass at CPA extending into IAC fundus	Normal	VS	R retrosigmoid craniectomy for tumor resection	CN VIII was unusually enlarged in the superior portion. The lesion was the same color as nerves, firmly attached to CN VIII in the cisternal portion; biopsy of the superior portion; facial stim triggered anteriorly and inferiorly.	Uncomplicated postop course with no facial weakness and intact hearing
Present Case	57 / M	R HL, dizziness, HA	MRI: non-enhancing mass in dilated IAC	Slight R > L SNHL	VS	Observation, followed by R retrosigmoid craniectomy for progressive symptoms	Intact CN VII and VIII complexes with evidence of the tumor located anterosuperiorly to the facial and cochlear nerve. Mass similar in appearance to neural tissue.	Preserved facial nerve function. Evidence of GTR on follow-up MRI.

The most common preoperative diagnoses, based on imaging, were vestibular schwannomas (n=6/8) [4,6-9], and one of these cases also included facial nerve schwannoma in the differential. [6] Preoperative differential diagnosis was not specified in two cases [3,5]. Management was generally surgical intervention for resectioning hearing or facial nerve function preservation (n = 8/8) [1,3-9]. Two of these cases were initially managed with observation but pursued surgical intervention for an increase in size [3] or a new CN deficit [6]. Histologic findings are described in Table 2. Most of these lesions were defined as purely glioneural hamartomas (n = 7) [3,5,7,9,11], but few cases also reported ganglionic elements (n = 3) [6,8,10], fibro adipose elements (n=1) [8] or specifically heterotypic cerebellar tissue (n=1) [4]. Outcomes were generally good, with stable to improved preoperative CN VII or VIII function and no evidence of tumor recurrence at follow-up (reported to range from five months to three years, n = 5) [3,4,6,7,9].

**Table 2:** Summary of reported histologic findings Abbreviations: GFAP = glial fibrillary acidic protein; PAS = periodic-acid Schiff

Author & Year	Reported Histologic Findings	Immunohistochemical Stains	Final Diagnosis
Babin et al., 1980 [10]	Normal-appearing astrocytes and neurons, no evidence of inflammation, glial reaction, or compression of adjacent structures; absence of invasion or compression;	PAS staining: sharp demarcation of myelinated nerve fibers from hamartoma	Neuronal and glial hamartoma
	Mature ganglion cells, astrocytes, and Rosenthal fibers	N/A	Neuronal and glial hamartoma
Ho et al., 1981 [11]	Normal-appearing astrocytes with dense neuroglial fibers and several hyalinized vessels. Rosenthal fibers and corporea amylacea. Scattered neurons of variable size and shape, some normal, others degenerated. Enclosed by a delicate sheath of collagen. No cellular features to suggest neoplasm.	N/A	Ganglionic hamartoma and neuroglial heterotopia
Palmer et al., 1996 [7]	Glial background with numerous large neurons containing abundant cytoplasm, Nissl substance and numerous processes. No satellite cells. Background of scattered glial filaments and occasional reactive astrocytes.	Bodian silver stain with normal in appearance.	Glioneural hamartoma
Hung et al., 1997 [6]	Frozen: Myelinated nerve fibers, intersected by fibrous tissue and normal-appearing neurons; Routine: Fascicles of normal myelinated axons intersected by fibrous and fibroadipose tissue septae, among which were observed mature ganglion cells containing well-formed Nissl substance, each with a single large nucleus and nucleolus. no atypical or binucleated neurons. some, but not all, of the ganglion cells were encircled by satellite cells. no areas of overt schwannoma or neurofibroma were present.	Synaptophysin, neurofilaments, S-100 with normal pattern of immunoreactivity in the neuronal cell bodies, axons and schwann cells; MIB-1 = 0%.	Hamartoma with ganglion cell, nerve and fibroadipose tissue
Carlvaho et al., 2003 [4]	Ganglionic cells, fascicles of nerve fibers, and fat tissue are irregularly distributed in a collagenous matrix.		Glioneural (ectomesenchymal hamartoma)
	Cross-striated muscle fibers, isolated nerve fibers, ganglionic cells, fat vacuoles, collagen.		Glioneural (ectomesenchymal hamartoma)
Goda et al., 2003 [5]	Concurrence of neuronal and non-neoplastic glial cells. Part of the tissue contained a molecular granular layer and medullar of the cerebellum but lacked a Purkinje layer and contained calcifications.		Cerebellar heterotopia
Ahmad et al., 2010 [3]	Normally myelinated axon fascicles are separated by fibrous stroma and adipose tissue. Along axons were multiple foci of mature ganglion cells and well-formed Nissl bodies in the peripheral cytoplasm. Foci of less mature ganglion cells.	Synaptophysin, neurofilaments, S-100 were normal; MIB-1 = 0%	Ganglionic hamartoma
Rizk et al., 2013 [8]	Frozen: neuroglial tissue; Routine: neuroglial tissue against a neurofibrillary background. Pale-blue ovoid deposits are compatible with corporea amylacea.	Vimentin, cytokeratin, GFAP, S-100 positive against a neurofibrillary background	Neuroglial heterotopia
Peris-Celda et al., 2018 [9]	Frozen: glioneural hamartoma; Routine: mature gray matter in which multiple large neurons identified	Luxol-blue/PAS stains: myelinated fibers running in fascicles and separating clusters of neurons	Glioneural hamartoma
Present Case	Glioneuronal tissue is consistent with hamartomatous brain tissue. Small fragments of nerve were included.	Neurofilament stain highlighted large neuronal cell bodies, some in clusters and intervening axons.	Glioneuronal hamartoma (or heterotopia).

The most common mass lesions in the IAC and cerebellopontine angle are neoplastic, with 80-90% being VS [1,2]. The next most common lesions after VS include meningiomas and epidermoids [1]. Hamartomas, conversely, are non-neoplastic lesions, representing an abnormal arrangement of tissue typically found in an organ system. These lesions are sporadic in the IAC and cerebellopontine angle.

Although rare, they may present with symptoms similar to their more common counterparts of auditory and vestibular dysfunction. This was how the majority of patients in the review of the literature, as well as in the present case, had presented. Unlike the neoplasms in this location, hamartomas are generally isodense with brain parenchyma. Interestingly, in our review of the literature, a slight majority of cases (n = 5/8) reported enhancement of the lesion [3,7,8], and 1 case reported minimal enhancement [9]. In the present case, no enhancement of the IAC lesion was noted. No cases, including ours, reported systemic involvement, and all lesions appeared in isolation.

Management of these lesions should be tailored to each patient. Asymptomatic or incidentally identified small lesions may be monitored, given the benign nature of glioneural hamartomas. Conversely, atypical or symptomatic lesions may warrant surgical resection to diagnose and preserve CN function. The surgical approach should be up to the discretion and comfort level of the surgeons as well as dependent on the patient's anatomy. In all cases with reported post-surgical outcomes, including ours, facial nerve and auditory nerve function were preserved [4,6-8], even though one case reported a transient postoperative facial nerve palsy that improved after 1 year [3]. No cases in the literature (n = 7/7) reported lesion recurrence on follow-up, as expected with this diagnosis [3-9].

## Conclusions

Glioneural hamartomas are rare non-neoplastic lesions found in the IAC, causing symptoms related to mass effect on the CN VII/VIII complex, but can be safely managed with preservation of CN function and low risk of recurrence. These cases are important to note as they are not common lesions but can cause significant CN deficits secondary to their location. Additionally, they may be easier to recognize once they are already symptomatic, given their slow-growing nature and the lack of consistent enhancement on imaging.
